# OffsampleAI: artificial intelligence approach to recognize off-sample mass spectrometry images

**DOI:** 10.1186/s12859-020-3425-x

**Published:** 2020-04-03

**Authors:** Katja Ovchinnikova, Vitaly Kovalev, Lachlan Stuart, Theodore Alexandrov

**Affiliations:** 1 Structural and Computational Biology Unit, European Molecular Biology Laboratory, Heidelberg, Germany; 2 Metabolomics Core Facility, European Molecular Biology Laboratory, Heidelberg, Germany; 3 Skaggs School of Pharmacy and Pharmaceutical Sciences, University of California, San Diego, La Jolla, CA USA

**Keywords:** Imaging mass spectrometry, Off-sample images, Pattern recognition, Machine learning, Deep learning, Artificial intelligence, METASPACE

## Abstract

**Background:**

Imaging mass spectrometry (imaging MS) is an enabling technology for spatial metabolomics of tissue sections with rapidly growing areas of applications in biology and medicine. However, imaging MS data is polluted with off-sample ions caused by sample preparation, particularly by the MALDI (matrix-assisted laser desorption/ionization) matrix application. Off-sample ion images confound and hinder statistical analysis, metabolite identification and downstream analysis with no automated solutions available.

**Results:**

We developed an artificial intelligence approach to recognize off-sample ion images. First, we created a high-quality gold standard of 23,238 expert-tagged ion images from 87 public datasets from the METASPACE knowledge base. Next, we developed several machine and deep learning methods for recognizing off-sample ion images. The following methods were able to reproduce expert judgements with a high agreement: residual deep learning (*F*1-score 0.97), semi-automated spatio-molecular biclustering (*F*1-score 0.96), and molecular co-localization (*F*1-score 0.90). In a test-case study, we investigated off-sample images corresponding to the most common MALDI matrix (2,5-dihydroxybenzoic acid, DHB) and characterized properties of matrix clusters.

**Conclusions:**

Overall, our work illustrates how artificial intelligence approaches enabled by open-access data, web technologies, and machine and deep learning open novel avenues to address long-standing challenges in imaging MS.

## Background

Imaging mass spectrometry (imaging MS) emerged as a powerful and versatile technology for spatial molecular analysis [3, 5, 6] with a particular interest in clinical and pharma applications [17, 20]. The capacities and potential of imaging MS were boosted with the introduction of high- and ultrahigh- resolving power mass spectrometry analyzers such as FTICR (Fourier-transform ion cyclotron resonance) and Orbitrap that rapidly shifted the current focus towards applications in spatial metabolomics and lipidomics [14]. Among various flavors of imaging MS, matrix-assisted laser-desorption ionization MALDI-imaging is the most widespread [14]. MALDI-imaging requires applying the so-called matrix onto the tissue section leading to formation of a layer of crystals covering the sample to facilitate “soft” energy transfer to sample analytes, enhance their ionization, and reduce in-source fragmentation [8]. However, the addition of ionizable matrix molecules contaminates the data with matrix ion signals which are not relevant for the molecular content of the sample and, moreover, can be isomeric or isobaric with sample analytes and thus mask out their spatial distribution. The matrix signals cannot be eliminated from the data with a simple approach, e.g. by considering [M + H] + matrix ions since a matrix forms hundreds of so-called matrix clusters, namely ions composed of matrix molecules. The presence of matrix cluster ions pollutes MALDI-imaging data by adding biologically irrelevant signals. This confounds quality control and statistical analyses, leads to false hits in metabolite identification and hinders downstream pathways analysis. Despite these negative effects being widely recognized, currently there is no solution for filtering out the matrix signals as their formation in general as well as for specific MALDI matrices, sample preparation protocols, and types of samples is poorly understood. A combinatorial model for matrix clusters was proposed [9] which suggests thousands of theoretically possible clusters whereas only some of them are observed in real experiments.

An expert can recognize a matrix ion image upon visual examination. A typical matrix image exhibits the so-called “background” or “off-sample” pattern with high intensities in the area outside of the sample and low intensities within the sample area due to the ion suppression effect [12, 19]. Manual filtering of off-sample ion images is not feasible due to the sheer size of an imaging MS dataset containing 10^4^–10^7^ ion images representing at least 10^2^–10^3^ molecules [15]. To the best of our knowledge, no automated methods for recognizing off-sample ion images were published [2]. presents a semi-automated method which requires manual selection of the off-sample area and some parameters and provides no evaluation or error analysis. Various software packages for imaging MS provide co-localization methods which in principle can be used for off-sample recognition but were never evaluated for this specific task. This gap likely persists due to the lack of a gold-standard dataset for evaluation of potential methods.

Artificial intelligence is a boosting field of research that already delivered numerous breakthroughs in image analysis in particular in image recognition. In this paper, we present an artificial intelligence approach to recognize off-sample mass spectrometry images. The approach capitalizes on using open-access data from the METASPACE knowledge base which was created by us [15] and populated by over 3000 public submissions from various labs (http://metaspace2020.eu). We shared a large number of mass spectrometry images from public METASPACE datasets with experts who tagged them as either on- or off-sample. We curated and integrated their judgements into a high-quality gold standard set. We developed several machine and deep learning algorithms for recognizing off-sample images. We trained and evaluated them on the gold standard and performed error analysis. In a test-case study, we investigated the off-sample images corresponding to the most popular MALDI matrix (2,5-dihydroxybenzoic acid, DHB) [18] and derived properties of matrix clusters using a cluster-generating combinatorial model. We have implemented the best method as a filter in the METASPACE free cloud platform. Overall, our work illustrates how artificial intelligence approaches enabled by open-access data, web technologies, and machine and deep learning open novel avenues to address long-standing challenges in imaging MS.

## Results

### Datasets selected for the gold standard

**Fig. 1:**
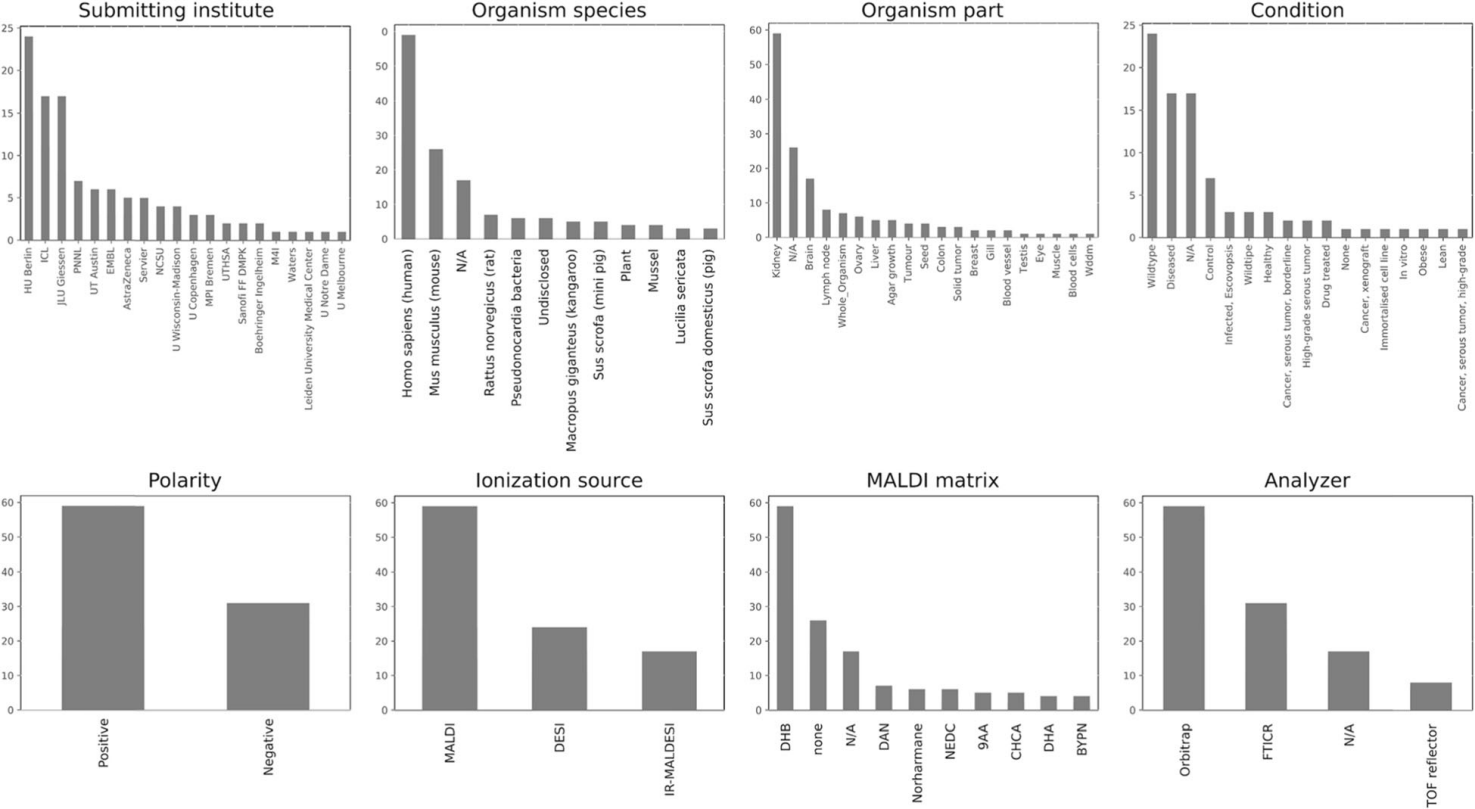
Properties of the public METASPACE datasets selected for the gold standard. They represent a variety of labs, types of samples, and technologies. Following a stratified random selection, this gold standard set reflects the full set of public datasets in the METASPACE knowledge base and, taking into account the size of METASPACE currently encompassing over 3000 public datasets, can be considered to be a representative sample in the field of imaging MS

**Fig. 2:**
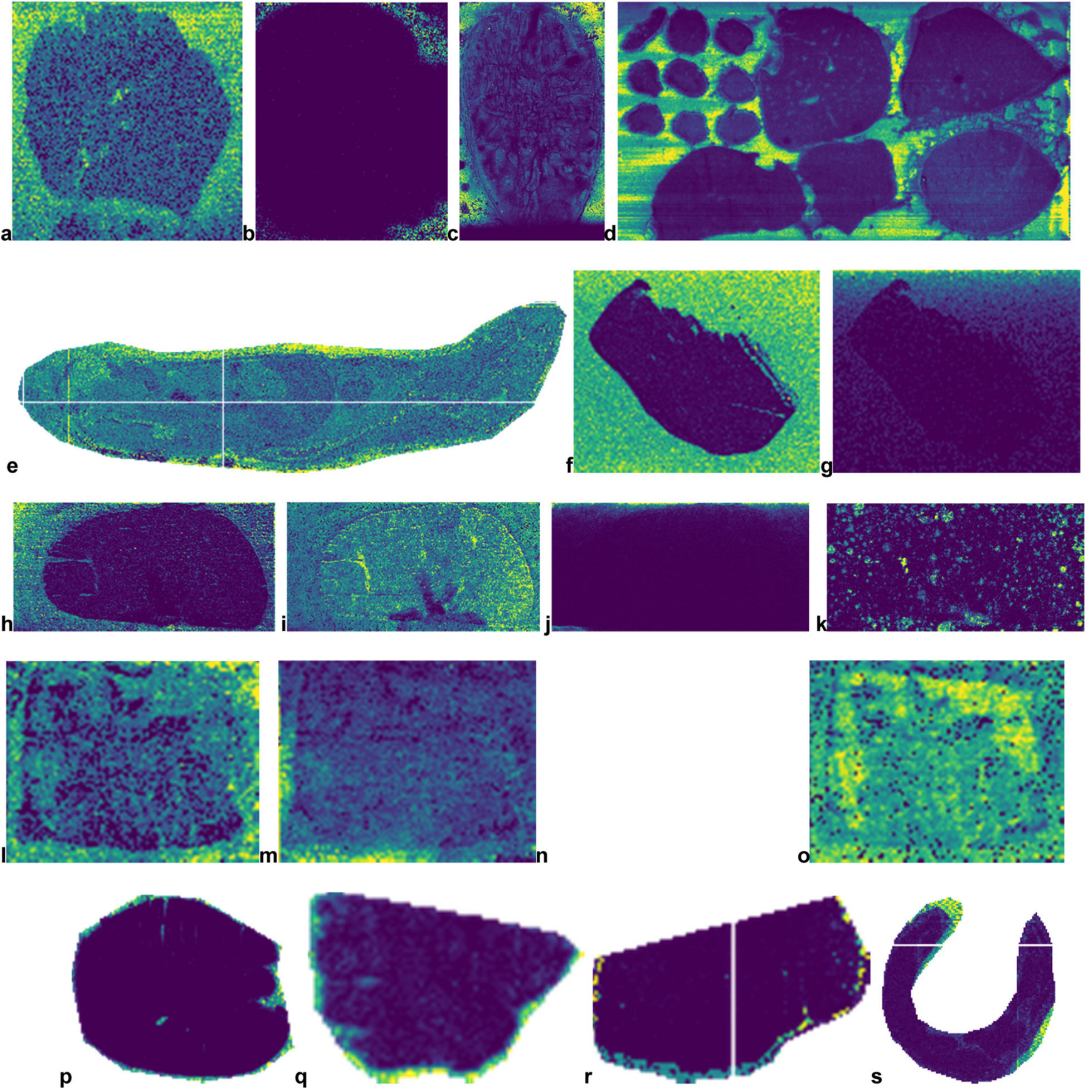
Representative off-sample ion images from the gold standard datasets illustrating a variety of spatial patterns exhibited by such images as well as particular aspects of the imaging MS datasets. **a**-**c**: Illustrative images from several datasets showing the indicative off-sample pattern (high intensities outside of the tissue section) from DESI- (**a**) and MALDI-imaging (**b**-**c**). **d**: A dataset with several tissue sections. **e**: A non-rectangular dataset where the acquisition area was selected around a whole body mouse tissue section. **f**: The indicative off-sample pattern. **g**: A gradually-changing off-sample pattern. **h**-**k**: Different spatial patterns of off-sample images in the same dataset (MALDI-imaging, DHB matrix). **h**: The indicative pattern. **i**: Everywhere-pattern. **j**: Gradually-changing pattern. **k**: Spotty pattern with spots everywhere, potentially due to a special matrix (DHB) application. **l**-**o**: Different spatial patterns of off-sample images in the same dataset (DESI-imaging). **l**: Mainly off-sample localization. **m**: Gradual off-sample localization. **n**: Off-sample leakage pattern. **o**: Everywhere pattern. **p**-**s**: Examples of off-sample MALDI ion images with only a narrow band of off-sample pixels due to the acquisition area selected precisely around the tissue section. METASPACE links to the ion images: **a**, **b**, **c**, **d**, **e**, **f**, **g**, **h**, **i**, **j**, **k**, **l**, **m**, **n**, **o**, **p**, **q**, **r**, **s**. For acknowledgements for these and other gold standard datasets, see section Acknowledgements

For the gold standard set, we selected 87 public imaging MS datasets from METASPACE (Supplementary Data D3). The datasets span various technologies and samples (Fig. 1). Importantly for the methods development, the gold standards includes datasets with both rectangular and non-rectangular imaged area. Also, some datasets included several tissue sections in the imaged area. For examples of representative off-sample ion images from the gold standard datasets see Fig. 2.

### Pilot study on creating a gold standard

The pilot study helped us learn about a variety of spatial patterns in off- and on-sample images (Fig. 2, Supplementary Data D1) as well as about the complexity and subjectivity of the tagging task, pitfalls of using automated strategies, as well as estimate resources necessary to obtain a sufficiently large gold standard set of tagged ion images. Moreover, we understood the importance of avoiding semi-automated tagging in order to avoid potential biases. This required implementing a web app for tagging, as simple approaches not requiring any special software are not feasible for obtaining a sufficient number of tagger ion images.

### Gold standard

The obtained gold standard includes 23,238 manually tagged ion images, of them 13,329 “off-sample” and 9909 “on-sample”, available at https://github.com/metaspace2020/offsample. The ion images tagged as “unknown” were not included into the gold standard.

### Agreement between taggers

Supplementary Table S1 shows the average pairwise inter-tagger agreement values for each of the five selected datasets. Note that those datasets, in curator opinion, were among the most difficult datasets to tag. The tagger-curator agreement values range from 0.89 to 1.0 with the average of 0.97 and inter-tagger average agreement values ranging from 0.71 to 0.98 with the average of 0.89. We speculate that the dataset “Servier_Ctrl_mouse_wb_median_plane_DHB” was likely hard to tag due the whole-body section including organs of various properties (density, ionization) as well as presence of the skin-localized ions which resemble matrix ions with high intensity at the perimeter of the section (Supplementary Figure S2). Next, we evaluated the performance of each tagger individually, and their agreement with other taggers. Supplementary Table S2 shows that taggers highly agree with the curator (with the Cohen κ greater than 0.95). The opposite would indicate excessive curation that could be associated with a bias introduced by the curator. Tagger2, Tagger3, and Tagger4 show a strong inter-tagger agreement (Cohen κ greater than 0.92), while Tagger1 and Tagger5 show a moderate inter-tagger agreement (Cohen κ 0.74 and 0.88).

To provide an additional validation of the achieved high inter-tagger agreement, we also calculated Krippendorff’s alpha. The average Krippendorff’s alpha is equal to 0.9 similar to Cohen’s κ (0.89); see Supplementary Table S1.

### Performance of the off-sample recognition methods

#### Spatio-molecular biclustering method

The results of the unsupervised spatio-molecular biclustering method are shown in Supplementary Table S3. The biclustering method performed the best when combined with the SVM (support vector machine) with linear kernel, among other machine learning methods. The optimal values of the method parameters for cluster assignment were *cluster_percent* = 0.09, *border_percent* = 0.1, *full_percent* = 0.1. We investigated the datasets for which the method performed poorly, i.e. the *F*1 score per dataset for either the “off-sample” or “on-sample” class was below 0.7, which accounted to eight datasets in total. We visually inspected the spatial clusters for each dataset. For three out of eight datasets, the spatial clusters were visibly noisy (e.g. Supplementary Figure S1a) as compared to visibly homogeneous clusters for the remaining 84 datasets (e.g. Supplementary Figure S1b). For two out of poorly performed eight datasets, we observed the wrong assignment of large spatial clusters to either “off-sample” or “on-sample” class (Supplementary Figure S1c-d). We additionally examined all other datasets and confirmed that for all but these two datasets, the assignment was correct.

In order to improve the method, we considered a semi-automated strategy when a user would curate the assignment of two largest spatial clusters. This semi-automated method is easy to implement as the curation can be performed just once for a dataset, and requires a quick visual examination and one click for assignment of one of the two spatial clusters to the “off-sample” class. For the semi-automated method, we manually swapped labels for two datasets for which automated assignment failed (Supplementary Figure S1c-d) that improved the performance up to the *F*1 score of 0.96 for off-sample and 0.97 for on-sample (Supplementary Table S3, second row).

For 78 out of 87 datasets, the method produced two large spatial clusters per dataset. For the remaining nine datasets, two large clusters and, in addition, from one to eight smaller spatial clusters per dataset were found. In total, 14 out of 16 of the small clusters were assigned correctly.

#### Molecular co-localization method

Similar to the bi-clustering method, the molecular co-localization method performed the best when combined with the SVM with the linear kernel. The performance is shown in Supplementary Table S4 (first row). Since the molecular co-localization method maps all ion images into the molecular space of all molecular formulas annotated at FDR (false discovery rate) < =50%, it is natural to assume that this method works the best when applied to datasets of similar molecular content. To test this hypothesis, we applied it only to a group of MALDI-imaging datasets acquired using the 2,5-dihydroxybenzoic acid (DHB) matrix in the positive ion mode. The polarity mode and the MALDI matrix are among the most influential parameters determining the molecular content, as molecules have preferential ionization polarity and affinity to a MALDI matrix. Supplementary Table S4 (second row) shows that indeed, the performance of the classifier when applied to DHB data only has been improved, particularly for recognizing off-sample images with the *F*1-score increased from 0.9 to 0.96 (though not significant considering the confidence intervals of +/− 0.06).

#### Comparing performance of all methods

Table 1 compares the performance of all developed methods for recognizing off-sample ion images. Since our intended application was filtering out off-sample ions, we were mostly interested in the high *F*1 values for off-sample detection. The best performing method was the deep residual learning that achieved the *F*1 off-sample score of 0.97 +/− 0.01. This method had high stable recall and precision values for off-sample detection (0.98 +/− 0.03 and 0.96 +/− 0.03), while its detection of on-sample was less reliable (0.94 +/− 0.07). The second best method with the *F*1 score of 0.96 +/− 0.03 for off-sample was the semi-automated spatio-molecular biclustering where curation of the cluster assignments to off/on classes was performed for two datasets. This method proved to be the most reliable for on-sample detection with *F*1 equal to 0.97 +/− 0.01.The spatio-molecular biclustering method without any curation achieved the *F*1 score of 0.93 +/− 0.1 for off-sample and 0.95 +/− 0.06 for on-sample. Note that the biclustering method is unsupervised and the gold standard was used only to select the parameters. The off-sample *F*1 score of the molecular co-localization method was 0.90 +/− 0.07 only. Note that the molecular co-localization method performed considerably better (*F*1 score of 0.96 +/− 0 .06) on a reduced set of DHB positive data (Supplementary Table S4) that potentially indicates that molecular co-localization can perform on par with other methods when applied to data collected with the same matrix. Supplementary Data D1 describes the performance of the template-image method which achieved the *F*1 score of 0.96 +/− 0.06 and 0.92 +/− 0.14 in semi- and fully-automated scenarios, respectively (Supplementary Table S6).

**Table 1:** Performance of the developed methods for recognizing off-sample ion images as evaluated on the gold standard of 23,238 ion images showing *F*1-score (*F*1), precision (*P*), and recall (*R*). For each measure, we show the average and confidence intervals (+ − two standard deviations) over five folds of the cross validation

	off-sample			on-sample		
	*F*1	*P*	*R*	*F*1	*P*	*R*
Deep residual learning	.97 (+/−.01)	.96 (+/−.03)	.98 (+/−.03)	.96 (+/−.04)	.97 (+/−.03)	.94 (+/−.07)
Semi-automated spatio-molecular biclustering, clusters curated for 2 datasets	.96 (+/−.03)	.96 (+/−.07)	.96 (+/−.04)	.97 (+/−.01)	.97 (+/−.03)	.97 (+/−.03)
Spatio-molecular biclustering	.93 (+/−.10)	.92 (+/−.10)	.94 (+/−.11)	.95 (+/−.06)	.95 (+/−.06)	.95 (+/−.06)
Molecular co-localization	.90 (+/− .07)	.95 (+/− .08)	.86 (+/− .15)	.93 (+/− .05)	.91 (+/− .11)	.96 (+/− .07)

### Off-sample results for specific types of imaging mass spectrometry

#### MALDI imaging with the DHB matrix

Recognition of off-sample ion images can have numerous applications and generally is expected to improve downstream data analysis. Here, we applied it to characterize the properties of DHB matrix clusters. First, we selected 31 MALDI-imaging datasets from the gold standard which were acquired using the DHB matrix in the positive ion mode; see Supplementary Data D4. Then, 353 molecular formulas of the DHB matrix clusters were generated following a combinatorial model proposed earlier [9]. We then created a custom molecular database for METASPACE by combining these molecular formulas with the HMDB (Human Metabolome Database) v4 database [21] and re-annotated the 31 datasets using this database.

For the 31 selected datasets, 6677 ions of DHB matrix clusters were annotated by METASPACE with an FDR < =50%. Of them, 3134 (47%) were recognized as off-sample and thus can be confidently associated with the matrix. These ions represent approximately 5% of all 58,203 annotated ions for the selected datasets. Per dataset, we recognized from zero (for one dataset only) to 314 off-sample DHB matrix cluster ions (101.1 ions on average). This quantifies the amount of biologically-unrelated information that should be removed prior to any downstream analysis.

From the recognized matrix cluster ions, 4.3% of them are isomeric with non-matrix molecules in HMDB v4. This quantifies the ambiguity of metabolite annotation which turns out to be relatively low.

**Fig. 3:**
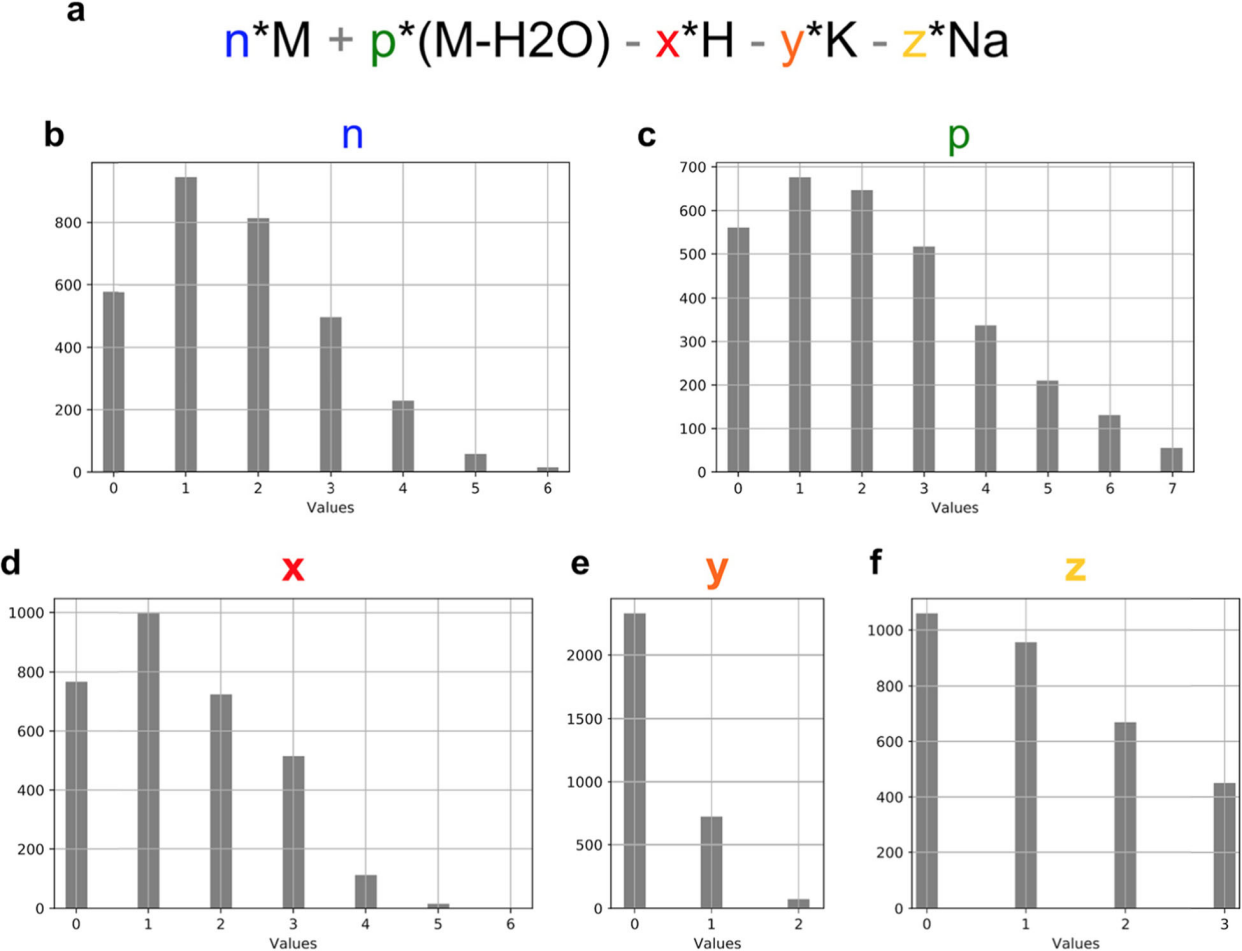
Parameters of the recognized DHB clusters. **a**: The formula n*M+p*(M-H2O)-x*H+y*K+z*Na represents the combinatorial model for matrix clusters from (Keller and Li, 2000) with M representing the DHB matrix molecular formula (C_7_H_6_O_4_). **b**-**c**: Histograms of parameters of the formula from A among ions in 31 MALDI-imaging DHB positive mode datasets which were annotated by METASPACE with an FDR<=50% and ion image recognized as off-sample

Figure 3 shows the parameters of the recognized matrix clusters. Interestingly, the recognized clusters exhibit broad patterns for all individual components of the combinatorial model “n*M + p*(M-H2O)-x*H + y*K + z*Na” [9] with a slight prevalence to form clusters including two molecules of DHB. Supplementary Table S5 shows the most frequently recognized matrix clusters; for a full list, see Supplementary Data D4. Two most frequent clusters were recognized in 28 out of 31 datasets. Note that the protonated ion of the intact DHB matrix, [M + H]+, the ion that one would commonly expect to be detected, was recognized only in 11 datasets (35% of all datasets). Interestingly, among the most frequently detected matrix clusters (Supplementary Table S5) there are no clusters containing potassium (K). This is also visible in Fig. 3e which shows that for a prevalent majority of recognized DHB cluster ions the parameter “y” corresponding to the inclusion of potassium in the cluster ion is equal to 0. The most frequent matrix cluster with potassium was “2*M + 1*(M-H2O)-1*H + 1*K + 0*Na”, detected in 14 datasets (45% of all datasets).

#### DESI imaging

We have applied the best off-sample classifier to all DESI (desorption electrospray ionization) imaging datasets from the gold standard (see Supplementary Data D5 as well as all images in section “Gold standard ion images predictions” at https://github.com/metaspace2020/offsample). Among all molecules formulas detected in METASPACE with FDR of at least 50%, ions with two molecular formulae were classified in more than half of the datasets (in datasets from two labs: Takats lab from Imperial College London and Everlin lab from UT Austin: C18H30O3S (in 14 out of 24 datasets) and C17H28O3S (in 13 out of 24 datasets). Interestingly, some off-sample DESI ion images show “glowing” or “border” spatial patterns with high intensities around the border of the tissue section (see Supplementary Figure S4).

## Discussion

Machine learning, and in particular deep learning, has demonstrated its capacities to outperform other approaches in various fields of science and technology including image and speech recognition, computer vision, and artificial intelligence [11]. Currently, deep learning is making its way into computational biology [1]. However, for exploiting potential of machine learning algorithms, one needs annotated or tagged data for training and evaluation. When solving molecular questions in the context of imaging mass spectrometry, the annotated data is often hard to obtain due to the lack of ground truth information about molecular content of the analyzed sample. Here, we decided to explore the potential of machine and deep learning approaches to tackle a problem which on a small scale can be solved by an expert: recognition of off-sample ion images. Our expertise in creating a high-quality gold standard [13] together with the exploited techniques of modern web development helped create a high-quality gold standard of 23,238 images.

This work was enabled by METASPACE, an open knowledge base of imaging MS data [15]. We used public datasets from 20 laboratories with the goal to obtain a gold standard that would be representative for the current state of the art, and to create methods that would have a wide impact in applications involving common imaging MS technologies and types of samples. By making their data public on METASPACE, these labs and their members made this study possible. Public semi-structured data is becoming increasingly useful and, through enabling novel computational developments, will ultimately benefit the field of imaging MS.

The software technologies used in METASPACE are another cornerstone in this study. The tagging web app TagOff was created based on METASPACE. Moreover, the flexible technology behind METASPACE enabled us to create a custom molecular database including DHB matrix clusters to screen for them in tens of datasets in a short time. We also used METASPACE for interactive data visualization, and sharing data and images in this paper. As a future step, we are planning to integrate the developed recognition of off-sample ion images into METASPACE and make it available to all users and in particular to those who provided public data used for training classifiers.

Working with semi-structured data from various sources is challenging because of the lack of complete information about biological systems, sample preparation, data acquisition, and data pre-processing. METASPACE metadata captures essential parts of this information (https://github.com/metaspace2020/metadata). Here, we demonstrated that the problem of recognizing off-sample ion images can be successfully solved using minimal metadata.

Note that our approach does not solve the segmentation problem of finding pixels which are in the off-sample region. Our approach is aimed to find and filter out those ion images which correspond to MALDI matrix or other background interferences. Although its results can be used to infer off-sample region e.g. creating an average off-sample ion image and taking high-intensity regions, this task is beyond the scope of this study.

In this work, we compared classification approaches of different kinds: machine learning employing either molecular or spatio-molecular content as well as a computer vision approach based on deep learning. We discovered that deep learning outperforms other approaches in the *F*1 off-sample score, with a semi-automatic expert-driven method based on spatio-molecular biclustering almost matching and outperforming it in the *F*1 on-sample score. The usual drawback, also experienced by us when using various deep learning frameworks, is that deep learning networks require a substantial training time, namely days on specially-devoted GPU (graphics processing unit)-equipped power station than hinders optimization. However, a combination of using a modern and fast framework (fast.ai), advanced deep learning methodology (residual learning), and training optimization make it possible to outperform the best expert-driven approaches.

Finally, we have implemented the deep learning classifier in the METASPACE platform, accessible for as new filter (click at “Add filter”, then “Show/hide off-sample annotations”). The implementation is also available through the REST API (https://github.com/metaspace2020/metaspace/tree/master/metaspace/off-sample). We believe that this example of how open-source software can be improved by using public data, collective input from the users, and deep learning, represents a model how challenging problems can be solved in the age of machine learning and open-access data.

## Conclusions

We believe that this work will not only help solve an important problem in imaging MS, but also will serve as an example of creating a high-quality annotated gold standard and subsequently applying machine learning and deep learning methods in this field. The access to public semi-structured data allowed us to create a gold standard data which is representative for the field of imaging MS and to develop methods that will have a wide impact beyond just one laboratory or research group.

## Methods

### Creating a gold standard set of ion images

In image recognition, a gold standard set is a collection of images manually tagged by experts called taggers often curated to ensure the highest quality. Having a gold standard set enables training and evaluating machine learning algorithms. However, creating an unbiased, representative, and balanced gold standard is a substantial challenge on its own. To the best of our knowledge, there exists no gold standard set of off-sample images for imaging MS.

#### Selecting imaging MS datasets for the gold standard

To create a gold standard set of off-sample and on-sample ion images, we selected public datasets from METASPACE with the aim to have a manageable number of diverse yet representative sample of datasets from different labs, samples, and imaging MS methods. First, we randomly selected 100 datasets from the public datasets in METASPACE. We added 14 more manually picked datasets to increase the diversity of the set and to represent a particular lab, biological model, sample preparation protocol, imaging MS source or analyzer that were not picked by the random sampling. We excluded 27 datasets from one big METASPACE submitter to reduce the lab, technology, and sample type bias, which left us with 87 gold standard imaging MS datasets.

#### Pilot study

Before creating a gold standard, we ran a pilot study to investigate the difficulty of recognizing off-sample ion images, as well as to learn potential pitfalls and obstacles of the tagging process by involving two taggers; see Supplementary Data D1 for more information.

#### Web app for manual tagging of ion images

**Fig. 4:**
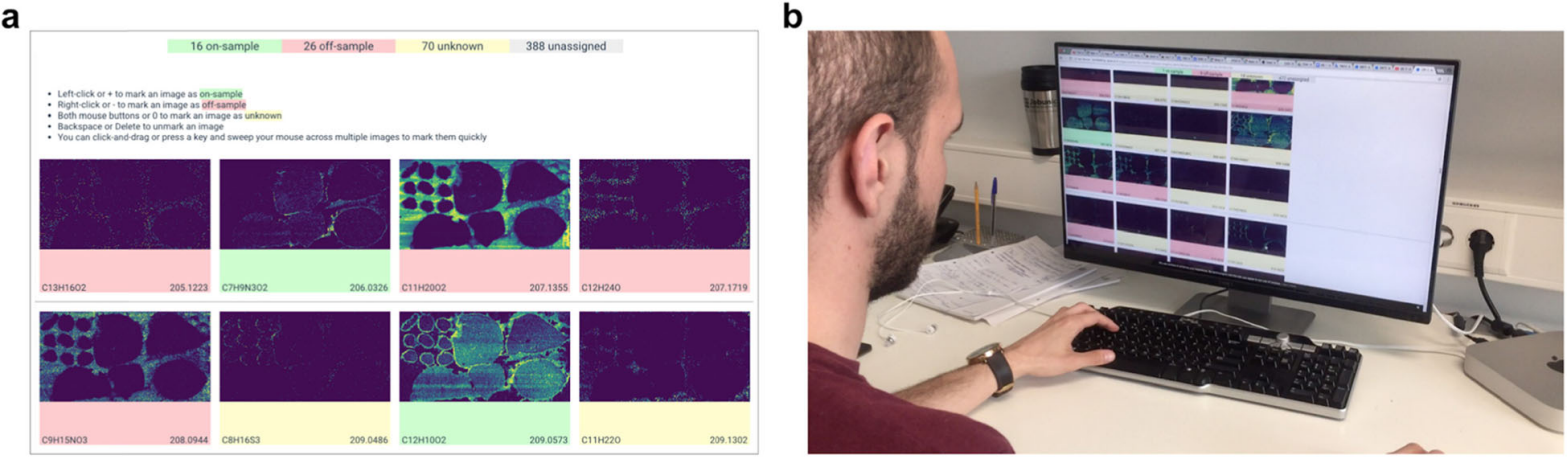
The TagOff web app for facilitated tagging off-sample ion images by using mouse clicks or keyboard shortcuts. **a**: The layout of the web page in TagOff. **b**: A tagger tagging ion images from a DESI-imaging dataset of five liver sections, contributed by Nicole Strittmatter, AstraZeneca (link)

The TagOff webapp (https://github.com/metaspace2020/offsample) was developed with the aim to facilitate tagging as well as help inspect tagged images, correct the tagging, and curate the gold standard. For a public dataset in METASPACE, the web app downloads ion images from METASPACE using the GraphQL API (link), shows ion images, and let a tagger tag each image into one of three classes (“off-sample”, “on-sample”, “unknown”). Tagging can be performed either by a single- or double-mouse click or, alternatively, by pressing shortcut keys while hovering the mouse. Also, we implemented a random subsampling that selects a specified number of ion images randomly; if a dataset has a lower number of ion images, all ion images would be considered. For each selected dataset and each tagger involved, we generated unique URLs containing the dataset and tagger names, and the maximal number of ion images to be considered. The tagging results are stored in real time, associated with the tagger name, and can be opened by either the same tagger or a curator; see Fig. 4, video in Supplementary Data D2.

#### Creating the gold standard set

For every gold standard imaging MS dataset, we considered annotations at a False Positive Rate < = 50% (the most permissive value in METASPACE) and randomly selected 500 ion images for tagging. For datasets with less than 500 ions annotated, all ion images were considered. We recruited five taggers with previous experience in mass spectrometry and explained to them the motivation behind the study and how to use the TagOff web app. No optical images or information about sample, imaging MS, protocols used to acquire the images were provided to the taggers. The taggers were instructed to spend less than 1 s per ion image. We also explained molecular and sample preparation factors contributing to generation of off-sample ion images (matrix application in MALDI, spraying in DESI), the ion suppression effect leading to lower intensities of matrix signals within the sample region, as well as advanced imaging MS effects which can affect how images look like (high noise, metabolite leakage, wet matrix application). Importantly, the tagging was performed before recognition methods were developed to ensure that taggers are not biased by knowing how methods work.

All 87 gold standard datasets were randomly distributed between five taggers, with each dataset assigned to one tagger. Each tagger received a set of pre-generated TagOff URLs. After the tagging process was completed, “off-sample” and “on-sample” tags in each dataset were reviewed by a curator with experience in imaging MS by quick skimming through images of the same class and re-tagging when necessary. The curation aimed to ensure that the taggers understood the task correctly and to minimally correct obvious mistakes. For two taggers, after seeing inconsistencies in the first tagged datasets, the curator instructed the taggers again and asked to repeat the tagging.

### Evaluating the gold standard

We assessed the complexity of the off-sample classification task and the reproducibility of the taggers judgements by calculating an inter-tagger agreement. For this, after the tagging was completed, the curator selected five gold standard datasets which were, in their opinion, among the most difficult datasets to tag. All taggers, who did not tag these datasets initially, were asked to tag them additionally, so that each of these five datasets was tagged by all five taggers. Then, we computed the taggers’ pairwise Cohen’s kappa agreement coefficient [4] for all ion images from these five datasets which were tagged as “off-sample” or “on-sample”. The images tagged as “unknown” by at least one tagger in the pair were ignored. We also computed Krippendorff’s alpha [10] ignoring “unknown” tags.

### Methods for automated recognition of off-sample images

We developed three methods for automated recognition of off-sample ion images. Supplementary Data D1 describes one more, template-image method.

#### Spatio-molecular biclustering method

The “spatio-molecular biclustering” method assumes that pixels of the off-sample area have similar molecular profiles as well as molecules corresponding to the off-sample area have similar distributions. First, for each dataset we found the border pixels. Note that, as discussed earlier, some of the considered datasets have non-rectangular calculated borders. Second, for each dataset we considered a matrix of ion intensities in all pixels. We performed biclustering using *SpectralCoclustering* from the scikit-learn v0.19.1. We iteratively increased the number of clusters from two to 20 until we found two large spatial clusters occupying more than *cluster_percent* (method parameter) percent of all pixels. Third, we assigned the resulting clusters to either “off-sample” or “on-sample” class. For the two large clusters, the cluster with a larger number of border pixels was assigned to “off-sample”; the other large cluster was assigned to “on-sample”. For a smaller cluster, the cluster was classified as off-sample if its border pixels occupied more than *border_percent* of all border pixels or more than *full_percent* of all small cluster pixels; otherwise it was assigned to “on-sample”. Biclustering of pixels and ions immediately provided assignment of ions to either “off-sample” or “on-sample”. Note that biclustering is an unsupervised method and requires no training. For selecting the parameters *cluster_percent*, *border_percent*, and *full_percent*, we used the five-fold cross validation. The resulting parameters were averaged over the five folds.

#### Molecular co-localization method

The “molecular co-localization” method assumes that off-sample ions are similar across datasets in terms of which ions they are co-localized with. We considered all ions annotated in at least one gold standard dataset with an FDR < = 50%. Within each dataset, we considered each ion as a vector of its intensities in all pixels. We represented all ions in a molecular space of all molecular formulas in all gold standard datasets as follows. First, we computed pairwise cosine similarities between all ions. Then, for each ion I and each molecular formula M (corresponding to a dimension of the molecular space), we considered the maximal similarity between the ion I and the ions M_n corresponding to the molecular formula M. Then, once the ions were mapped into this molecular space, we considered we considered a variety of classifiers from the scikit-learn Python package v0.19.1, including the Nearest Neighbors, linear SVM, RBF (radial basis function) SVM, Decision Tree, Random Forest, Naive Bayes, AdaBoost, QDA (quadratic discriminant analysis), Gaussian Process, Neural Net classifiers, and evaluated them on the gold standard images as described later in section “Classifiers evaluation”.

#### Deep residual learning method

In the last method, we exploited Deep Residual Learning, a recently introduced deep learning approach to image recognition [7]. The advantage of the residual learning is faster training of a network which at the same time is deeper than comparable networks. Residual learning was demonstrated to outperform other deep learning approaches in the tasks of image recognition and segmentation in several competitions in 2015. We used the fastai library (v1.0.34) with the PyTorch framework (v1.0) with the ResNet-50 model from the torchvision library (v0.2.1). First, all ion images of non-rectangular datasets were padded with zeros. Then, the images were normalized with the ImageNet statistics (per channel mean and std), resized and cropped to 224 × 224 and, per requirement of the model, converted into RGB three-channels images by pasting the same ion image into all three channels. In order to increase the training set, we applied data augmentation with the settings default for the fast.ai library that included the following transformations: image random crop/pad, horizontal and vertical flip, image warping, rotation, zooming, brightness and contrast adjustment. We used the ResNet-50 network pre-trained on the ImageNet images [16]. The training is described in detail in Supplementary Data D1. The validation was performed as described later. The trained deep learning model as well as a microservice implementation are available at https://github.com/metaspace2020/offsample.

### Classifiers evaluation

The considered classifiers were optimized to maximize the *F*1 score for the off-sample class. We also calculated the precision and recall to provide more detailed information about the performance. For each algorithm, we performed five-fold cross-validation when the whole gold standard was randomly split into five parts, each subsequently taken out for evaluation of the classifier trained on the rest of the data. To avoid overfitting, we used a grouped five-fold cross validation where ion images from the same dataset were all assigned either to the training or validation part.

## Supplementary information

**Additional file 1 **: **Supplementary Figures S1-S4**

**Additional file 2 **: **Supplementary Tables S1-S6**

**Additional file 3 **: **Supplementary Data D1-D5: D1:** “Supplementary methods and results.pdf”. **D2:** “Interactive tagging of ion images using web app.mov”, video of a tagger using the TagOff web app. **D3:** “Gold standard datasets.csv”, metadata of 87 public datasets from METASPACE selected for the gold standard. **D4:** “DHB matrix clusters frequencies.csv”, results of annotation of 31 gold standard datasets acquired using the MALDI DHB matrix and positive ion mode and off-sample recognition for DHB matrix clusters generated according to a combinatorial model. **D5**: “DESI offsample ions frequencies.csv”, a file showing for each molecular formula the number of DESI imaging datasets from the gold standard where ions with such molecular formula were classified as off-sample.

## Data Availability

The gold standard, source code for evaluation, deep learning model, results of the deep learning model for all ion images from the gold standard, and a microservice implementation with REST API are available at: https://github.com/metaspace2020/offsample.

## Abbreviations

DESI: Desorption electrospray ionization
DHB: 2,5-dihydroxybenzoic acid
FDR: False discovery rate
FTICR: Fourier-transform ion cyclotron resonance
GPU: Graphics processing unit
HMDB: Human metabolome database
MALDI: Matrix-assisted laser desorption/ionization
MS: Mass spectrometry
QDA: Quadratic Discriminant Analysis
RBF: Radial Basis Function
SVM: Support vector machine
