# Supplementary material for: OffsampleAI: artificial intelligence approach to recognize off-sample mass spectrometry images
Source: BMC Bioinformatics. 2020 Apr 3;21:129. doi: 10.1186/s12859-020-3425-x (PMC7119286; doi:10.1186/s12859-020-3425-x)
Supplement: Supplementary file 2 — Additional file 2 : Supplementary Tables S1-S6 [file 12859_2020_3425_MOESM2_ESM.pdf]

Supplementary Tables

Dataset	Tagger-curator average	Inter-tagger pairwise average
70%meoh_8cyc_75um	1.0	0.86
DESI quan_Swales	0.97	0.95
ICL//A51 CT S3-centroid	1.0	0.93
Mousebrain_MG08_2017_GruppeA	0.99	0.98
Servier_Ctrl_mouse_wb_median_plane_DHB	0.89	0.71
**Average**	**0.97**	**0.89**

Supplementary Table S1. Agreement between the taggers and the curator for the five selected datasets from the gold standard. The datasets were selected to be among the most difficult datasets to tag. Cohen's κ was used to estimate the agreement. The tagger-curator average shows how well the taggers agree with the curated tags. The inter-tagger pairwise average shows the average pairwise agreement between taggers without considering the curated tags.

	Tagger1	Tagger2	Tagger3	Tagger4	Tagger5
**Tagger-curator average**	0.95	0.95	0.99	0.97	0.96
**Inter-tagger average**	0.88	0.92	0.92	0.93	0.74

Supplementary Table S2. Detailed investigation of the five taggers with respect to their agreement with the curator as well as between each other. For each tagger, Cohen-kappa agreement of the tagger with the curator and average pairwise inter-tagger agreement is shown.

	off-sample			on-sample		
	**F1**	**P**	**R**	**F1**	**P**	**R**
Spatio-molecular biclustering	.93 (+/-.10)	.92 (+/-.10)	.94 (+/-.11)	.95 (+/-.06)	.95 (+/-.06)	.95 (+/-.06)
Semi-automated spatio-molecular biclustering, clusters curated for 2 datasets	.96 (+/-.03)	.96 (+/-.07)	.96 (+/-.04)	.97 (+/-.01)	.97 (+/-.03)	.97 (+/-.03)

Supplementary Table S3. Performance of the unsupervised spatio-molecular biclustering method. F1-measure (F), precision (P), and recall (R) were calculated on the gold standard of 23238 images. For each measure, we show the average and confidence intervals (+- two standard deviations) over five folds of the cross validation.

	off-sample			on-sample		
	**F1**	**P**	**R**	**F1**	**P**	**R**
Molecular co-localization method, full gold standard	.90 (+/- .07)	.95 (+/- .08)	.86 (+/- .15)	.93 (+/- .05)	.91 (+/- .11)	.96 (+/- .07)
Molecular co-localization method, DHB positive data	.96 (+/- .06)	.96 (+/- .08)	.96 (+/- .06)	.95 (+/- .09)	.95 (+/- .07)	.94 (+/- .12)

Supplementary Table S4. Performance of the molecular co-localization method. F1-score (F1), precision (P), and recall (R) are shown. The method was evaluated on the full gold standard of 23238 images, as well as on a reduced set of the gold standard MALDI-imaging datasets acquired using the 2,5-dihydroxybenzoic acid (DHB) matrix in the positive ion mode. For each measure, we show the average and confidence intervals (+- two standard deviations) over five folds of the cross-validation.

Matrix cluster	Molecular formula	Absolute frequency	Relative frequency
1*M+2*(M-H2O)-0*H+0*K+0*Na	C21H14O10	28	90%
1*M+2*(M-H2O)-1*H+0*K+1*Na	C21H13NaO10	28	90%
1*M+1*(M-H2O)-0*H+0*K+0*Na	C14H10O7	27	87%
1*M+1*(M-H2O)-1*H+0*K+1*Na	C14H9NaO7	27	87%
1*M+1*(M-H2O)-2*H+0*K+2*Na	C14H8Na2O7	27	87%
0*M+2*(M-H2O)-1*H+0*K+1*Na	C14H7NaO6	26	84%
0*M+4*(M-H2O)-0*H+0*K+0*Na	C28H16O12	26	84%
0*M+3*(M-H2O)-0*H+0*K+0*Na	C21H12O9	25	81%
1*M+3*(M-H2O)-1*H+0*K+1*Na	C28H17NaO13	25	81%
1*M+3*(M-H2O)-0*H+0*K+0*Na	C28H18O13	24	77%

Supplementary Table S5. Most frequently annotated and recognized DHB matrix clusters. In the matrix cluster formula, M stands for the molecular formula of the DHB matrix ($C_7H_6O_4$). The absolute/relative frequencies stand for the number/percentage of datasets (out of 31 selected gold standard datasets) in which a particular matrix cluster was annotated by METASPACE with an FDR <=50% with an ion image recognized as off-sample.

	off-sample			on-sample		
	**F1**	**P**	**R**	**F1**	**P**	**R**
Template-based method, 4 templates	.92 (+/-.14)	.93 (+/-.09)	.91 (+/-.20)	.95 (+/-.06)	.94 (+/-.10)	.96 (+/-.03)
Semi-automated template-based method, 4 templates	.96 (+/-.06)	.98 (+/-.02)	.94 (+/-.13)	.97 (+/-.04)	.96 (+/-.09)	.98 (+/-.01)
Semi-automated template-based method, 2 templates	.95 (+/-.07)	.97 (+/-.03)	.93 (+/-.14)	.96 (+/-.05)	.95 (+/-.09)	.98 (+/-.02)

Supplementary Table S6. Performance of the template-based classifier. F1-score (F), precision (P), and recall (R) were calculated on the gold standard of 23238 images. For each measure, we show the average and confidence intervals (+- two standard deviations) over five folds of the cross validation.
