# Supplementary material for: OffsampleAI: artificial intelligence approach to recognize off-sample mass spectrometry images
Source: BMC Bioinformatics. 2020 Apr 3;21:129. doi: 10.1186/s12859-020-3425-x (PMC7119286; doi:10.1186/s12859-020-3425-x)
Supplement: Supplementary file 3 — Additional file 3 : Supplementary Data D1-D5: D1: “Supplementary methods and results.pdf”. D2: “Interactive tagging of ion images using web app.mov”, video of a tagger using the TagOff web app. D3: “Gold standard datasets.csv”, metadata of 87 public datasets from METASPACE selected for the gold standard. D4: “DHB matrix clusters frequencies.csv”, results of annotation of 31 gold standard datasets acquired using the MALDI DHB matrix and positive ion mode and off-sample recognition for DHB matrix clusters generated according to a combinatorial model. D5: “DESI offsample ions frequencies.csv”, a file showing for each molecular formula the number of DESI imaging datasets from the gold standard where ions with such molecular formula were classified as off-sample. [file 12859_2020_3425_MOESM3_ESM.zip › Supplementary Data D1, Supplementary methods results.pdf]

Supplementary Data S1. Supplementary methods and results

Supplementary Methods

Pilot study description

Before creating a gold standard, we ran a pilot study to investigate the difficulty of recognizing off-sample ion images, as well as to learn potential pitfalls and obstacles of the tagging process. In the pilot study, we involved two taggers, PilotTagger1 and PilotTagger2. Each tagger used their own semi-automated strategy to tag ion images.

PilotTagger1 selected 19 public datasets from METASPACE. For each dataset, PilotTagger1 semi-automatically outlined the off-sample area by using a k-means clustering-based spatial segmentation. For each ion image, the tagger scaled its intensities to [0,1] and calculated the sum of the intensities within the off-sample area. Then, the tagger selected ion images with the highest values of this score as off-sample, and ion images with the lowest values of this score as on-sample.

PilotTagger2 selected 110 public datasets from METASPACE. For each dataset, PilotTagger2 picked a template ion image for the off-sample area and, for all other ion images, calculated the Spearman correlation with the template ion image. Images with the correlation above a manually selected threshold were selected as off-sample; images with the similarity score below a manually selected threshold were selected as on-sample. Based on their expert knowledge, PilotTagger2 curated all selected images by opening them in a file browser and removing those which looked to be wrongly assigned.

Training the deep residual network

For training of the deep residual network described in detail in Methods, section "Deep residual learning method", we used the binary cross entropy as the loss function with the batch size of 96 and weight decay (L2 normalization) of 0.1. The training included two stages: first the training the network head (classifier) then fine-tuning the network body (feature extractors/convolutional layers). In the first stage, max learning rate (lr) was 3e-3, with five epochs. In the second stage, for the first third of the network lr was 1e-5, for the second third of the network lr was 1e-4, for the third third of the network lr was 3e-4, with five epochs. The training was done using the one cycle learning policy (see https://arxiv.org/pdf/1803.09820, by Leslie N. Smith, US Naval Research Laboratory, Washington, DC, USA) where learning rate rapidly increases to its max value and then decreases to a small value during all epochs. For the network weight optimizer we used the Adam algorithm with the max learning rate described above. The hyperparameters were selected manually by splitting the gold standard into a validation and training sets, and finding the hyperparameters providing the best F1 score for the off-sample images recognition.

Template-image method description

The “template-based” method is based on the assumption that off-sample images within a dataset are co-localized and exhibit a relatively homogeneous distribution in the off-sample area. For each dataset, we automatically selected two template off-sample ions and two template on-sample ions. For this, we considered the border pixels of the dataset. Note that some of the considered datasets were acquired within an arbitrary (non-rectangular) area, likely to reduce the acquisition time, and thus have non-rectangular calculated borders. For each ion, we calculated the sum of its normalized intensities in the border pixels. Two ions with the maximum border intensity were selected as template off-sample ions. Two ions with the minimum border intensity were selected as template on-sample ions. We represented each ion image in the dataset as a vector of its intensities in all pixels. Then, we computed cosine similarities between each ion and the template ions. As a result, each ion was represented in the template space as a vector of the length four. Then, in this template space, we considered a variety of classifiers from the scikit-learn Python package v0.19.1, including the Nearest Neighbors, linear SVM, RBF SVM, Decision Tree, Random Forest, Naive Bayes, AdaBoost, QDA, Gaussian Process, Neural Net classifiers. We evaluated the classifiers on the gold standard images as described later in section “Classifiers evaluation”.

Supplementary Results

Pilot study outcomes and lessons learned

The pilot study included tagging of thousands of ion images by two taggers (PilotTagger1 and PilotTagger2) each following their own semi-automated strategies. This experience helped us learn about a variety of spatial patterns in off-sample and on-sample images (Figure 3), complexity and subjectivity of the tagging task, pitfalls of using automated strategies, as well as estimate resources necessary to obtain a sufficiently large gold standard set of tagged ion images.

Although most off-sample ion images exhibited the indicative off-sample distribution (high intensities in the off-sample area, low intensities in the on-sample area, e.g. Figure 3a-d,f), for some datasets their off-sample ion images displayed a surprising heterogeneity in spatial patterning. Some off-sample ion images showed a gradual change of intensities (e.g. Figure 3g,j) that can be explained by spatial charging or gradual changes in calibration or intensities observed for individual molecules. Some ion images exhibited a “metabolite leakage” pattern (low intensities in the off-sample area but with high intensities at the perimeter of the section, Figure 3n) which were particularly hard and ambiguous to assign to either off- or on-sample class. Some off-sample ion images exhibited unexplainable patterns e.g Figure 3k. Some datasets contained no clear off-sample region or only a very small region containing only a narrow band of pixels around the sample e.g. Figure 3p-s. This was observed mainly for the non-rectangular datasets where the acquisition area was selected precisely around the tissue section area.

We have encountered the following issue when tagging off-sample ion images using a semi-automated strategy. We considered two semi-automated strategies. The first strategy included ranking ion images

according to a particular measure and then selecting a set of images having measure values above a cutoff. The second strategy included finding a set of off-sample ion images automatically and then curating them by using a visual examination. Any of such strategies using a tagging algorithm introduce a potential bias into the gold standard. In case a classification algorithm resembles the tagging algorithm, this would lead to a misleadingly high accuracy when evaluated on such a gold standard set.

This consideration motivated us to turn to using fully-manual tagging for creating the gold standard. This required implementing a web app to facilitate tagging, as from the pilot study we learned that simple approaches not requiring any special software (e.g. manual arrangements of images into folders in a file browser or tagging of images in a spreadsheet) are not feasible for obtaining a sufficient number of tagger ion images for a gold standard for machine learning algorithms. From the pilot study, we have also learned that it is necessary to introduce the "unknown" tag because many ion images are hard to classify and assigning them to either "off" or "on" categories would be rather arbitrary and would reduce the quality of the gold standard.

Template-image method performance

For the template-image method, among considered classifiers applied to the low-dimensional representation of images in the space of cosine scores to the template ion images, the Support Vector Machine (SVM) classifier with the linear kernel showed the best performance. Supplementary Table S6 (first row) shows the performance with the F1-scores equal to 0.92 and 0.95 for the off-sample and on-sample recognition, respectively.

We investigated cases when the method performed poorly. As expected, the main issue was selection of wrong templates. After visual examination of all template ions for all 87 datasets, we have identified 20 datasets for which at least one of the four automatically selected templates was incorrect as compared to gold standard annotation.

In order to improve the method, we considered a semi-automated strategy when a user would curate the selected template images. This semi-automated method is easy to implement as the operation would need to be performed just once for a dataset. For the semi-automated method, we manually selected template ions for the 20 datasets for which automated selection failed. As expected, the semi-automated method performed considerably better (see Supplementary Table S6 below, second row). We also investigated a simpler semi-automated version when only two template ions (one off-sample and one on-sample template) would be selected by a user as this version would be user friendlier in a real-life applications. The semi-automated version with two templates (see Supplementary Table S6, last row) outperformed the automated version and showed only a slight drop of performance as compared to the semi-automated version with four templates.
